# Dihydropyrrolo[3,2‑*b*]pyrroles as Novel Conjugated Polymers: Leveraging Simplicity for Sustainability and Student Success

**DOI:** 10.1021/acs.accounts.6c00298

**Published:** 2026-06-13

**Authors:** Graham S. Collier

**Affiliations:** School of Polymer Science and Engineering, 5104 University of Southern Mississippi, Hattiesburg, Mississippi 39406, United States

## Abstract

The field of π-conjugated polymers and molecules has realized tremendous development across numerous technologies due to the ability to easily manipulate macromolecular properties through molecular/monomer design strategies. This synthetic tailorability has led to breakthroughs in power conversion efficiencies (PCEs) for organic photovoltaics (OPVs), maximizing conductivity and switching contrasts for redox-active polymers, and interfacing with aqueous and biological systems. While these achievements are impactful, concepts of green chemistry have often been overlooked, contributing to a trend in which increasing device performance is accompanied by increased synthetic complexity. Researchers studying conjugated polymers have increasingly recognized the importance of synthetic complexity and are exploring simplified structures and preparatory protocols. As such, it will be beneficial for the field to find modular, scalable, and tailorable building blocks that simplify the preparation of conjugated polymers without sacrificing properties and ultimately performance metrics.

Early career faculty face numerous challenges especially as it relates to choosing a research topic and nurturing it for success. The PI’s independent research career began in the height of the COVID-19 pandemic which brought on additional challenges and necessitated a flexible approach for engaging students in research with constrained resources. Simultaneously, we were thinking of ways we could impactfully contribute to the field of conjugated polymers and we decided to “keep it simple”. Conjugated scaffolds built around 1,4-dihydropyrrolo­[3,2-*b*]­pyrroles (DHPPs) became a focal point of our efforts because of their reported ease of synthesis and purification but noted tailorability. During our preliminary literature searches we found these DHPPs had only been studied as *molecular* systems but their attributes were appealing for (1) engaging students with little-to-no experience in meaningful research, (2) launching a new research group with limited resources, and (3) providing the field with a simple building block for conjugated polymers suited for optoelectronic applications. We started our research journey with one question: “*Can DHPPs be designed to participate in polymerizations to create a novel class of tailorable conjugated polymers with reduced synthetic complexity?”*

This Account highlights our efforts to simplify the synthesis of conjugated polymers, install labile linkages into these simple polymers to facilitate degradability, and establish structure–property relationships to develop DHPPs as high-contrast, color-controlled electrochromic materials. Through these efforts, DHPPs have emerged as useful building blocks to quantifiably reduce synthetic complexity commonly associated with conjugated polymers, create new degradable conjugated polymers, develop novel color-controlled electrochromes, and engage students in meaningful and impactful research. As such, this Account is not only a report on our scientific endeavors but also a reflective exercise that might be useful for other early career faculty, especially those starting at resource-limited institutions, as they initiate independent research programs.

## Graphical abstract

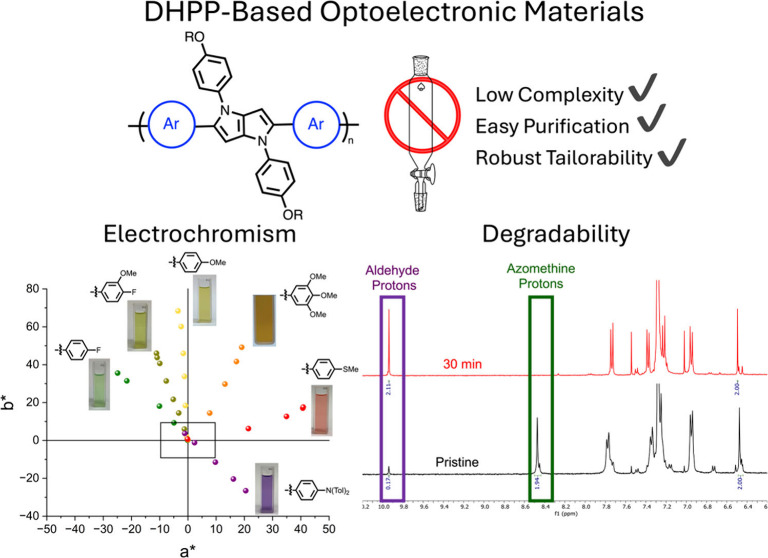

## Key References

-

Bell, K.-J. J. ; Kisiel, A. M. ; Smith, E. ; Tomlinson, A. L. ; Collier, G. S. Simple Synthesis of Conjugated Polymers Enabled via Pyrrolo­[3,2-*b*]­pyrroles. Chem. Mater. 2022, 34, 8729–8739 .^1^ This manuscript reports the first instance of DHPPs being directly enchained in the repeat unit of a conjugated polymer and establishing structure–property relationships of a new class of polymeric materials.
-

Bartlett, K. A. ; Charland-Martin, A. ; Lawton, J. ; Tomlinson, A. L. ; Collier, G. S. Azomethine-Containing Pyrrolo­[3,2-*b*]­pyrrole Copolymers for Simple and Degradable Conjugated Polymers. Macromol. Rapid Commun. 2023, 45, 2300220 10.1002/marc.20230022037449343.^2^ Installation of acid-labile azomethine groups in the backbone of DHPP-containing polymers enables attaining degradable DHPP polymers.
-

Bell, K.-J. J. ; Sabury, S. ; Phan, V. ; Wagner, E. M. ; Hawks, A. M. ; Bartlett, K. A. ; Collier, G. S. Synthesis of 1,4-Dihydropyrrolo­[3,2-*b*]­pyrrole-Containing Donor–Acceptor Copolymers and their Optoelectronic Properties. J. Poly. Sci. 2024, 62, 2975 .^3^ The structural design space of DHPP copolymers is expanded to using the donor–acceptor approach to manipulate optoelectronic properties leading to new electrochromic phenomenon and photovoltaic response of DHPP copolymers in device configurations.
-

Hawks, A. M. ; Daniel, L. M. ; Sorto, V. S. ; Mauro, J. ; Skiouris, P. ; Collier, G. S. Expanding Color Control of Anodically Coloring Electrochromes Based on Electron-Rich 1,4-Dihydropyrrolo­[3,2-*b*]­pyrroles. ACS Appl. Opt. Mater. 2024, 2, 1235–1244 38962565 10.1021/acsaom.4c00197PMC11217944.^4^ The structural tailorability of DHPPs enabled precise control of optical properties as neutral and oxidized species, leading to a new class of color-controlled, high-contrast electrochromic materials attainable in a single synthetic step.
-

Collier, G. S. ; Layton, J. T. ; Skiouris, P. ; Bell, K.-J. J. ; Wagner, E. W. ; Phan, V. ; Fraser, S. G. ; Tomlinson, A. L. Diversifying Peripheral Aromatic Units of Pyrrolo­[3,2-*b*]­pyrrole-Containing Conjugated Polymers and the Resulting Optoelectronic Properties. J. Mater. Chem. C 2025, 13, 8531–8543 .^5^ Repeat unit compositions of DHPP conjugated polymers are expanded to include thiophene and benzothiadiazole directly attached to the DHPP core such that optoelectronic properties are easily manipulated and redox stability is improved.

## Introduction

Conjugated polymers have been known since the 1800s but have received increased attention since they showed metal-like conductivities after doping, leading to the 2000 Nobel Prize in Chemistry.^6^ Over the next decades, there have been many iterations of “state-of-the-art” chemistries, structures, and device performance metrics that have enabled substantial advancement of the field. For example, the advent of controlled chain-growth polymerizations of alkylthiophenes was crucial for understanding effects of regiochemistry on polymer properties, sparked advances in catalyst development, and enabled understanding of fundamental processes in charge transport in organic photovoltaics (OPVs).^7,8^ Additionally, the arrival of the donor–acceptor design approach introduced new perspectives into effects of repeat unit composition on optoelectronic properties while pushing device performance metrics to new levels.^9–11^ The donor–acceptor design has also been a useful approach for spectral engineering of redox-active polymers used in electrochromic films and devices.^12^ The utilization of nonfullerene acceptors (NFAs) in the field of OPVs further pushed device metrics to new levels to where power conversion efficiencies (PCEs) are now approaching 20% for single junction devices.^13^ While each of these examples represent impactful advances for the field, each strategy introduces increased synthetic complexity such that while devices exceeded the expected performance threshold for commercialization, the costs associated with these increases is an additional factor that keeps conjugated polymers from being widely deployed. As such, there are examples of researchers shifting efforts to simplifying the preparation of conjugated polymers (i.e., reducing synthetic complexity) while maintaining device performance metrics.

With the desire to address the synthetic complexity issue with conjugated polymers, researchers ask; “*What does complexity mean?”* Over the past decade, groups that have turned some attention to simplifying the synthesis of conjugated polymers have used the equation derived by Po and co-workers (eq 1) to quantify the synthetic complexity (SC) for preparing new polymeric materials.^14^ The five variables in eq 1 are defined as the number of synthetic steps (NSS), the reciprocal yield of monomers (RY), the number of operations required for purification of monomers (NUO), the number of column chromatography purifications (NCC), and the number of hazardous materials used (NHC), all of which are assigned a weighted value based on the influence each step has on potential cost implications.

1 $$SC=35\frac{NSS}{{NSS}_{\max}}+25\frac{\log\left(RY\right)}{{\log(RY}_{\max})}+15\frac{NUO}{{NUO}_{\max}}+15\frac{NCC}{{NCC}_{\max}}+10\frac{NHC}{{NHC}_{\max}}$$

From eq 1, Po and co-workers calculated P3HT to have a SC of 7.8 while donor–acceptor copolymers reach values up to 92.4 for a π-extended benzodithiophene-based copolymer. McCulloch and co-workers have contended that discrepancies in hazard statements and differing interpretations of isolation vs purification are compounded when attempting to quantify a scalability factor (SF) for an OPV blend.^15^ As such, they proposed eq 2, where only the NSS and RY for the donor (D) and acceptor (A) are accounted for since they are most weighted variables in eq 1. The χ_D/A_ is the mass percent of each component and is also accounted for in these calculations.

2 $${SF}_{Blend}=\sum \chi_{D/A}\sum {SF}_{D/A}=\sum \chi_{D/A}({NSS}_{D/A}+{RY}_{D/A})$$

While these equations each represent valued additions to the philosophical debate the field is having, inherent biases exist based on what each researcher finds to be the most impactful contributor for SC, especially for OPV active-layer materials. Marks and co-workers analyzed fluorinated vs nonfluorinated analogs of high-performing building blocks as a test-base comparison to emphasize the delicate balance between increasing synthetic steps (via halogenation) and improving OSC performance metrics.^16^ Specifically, halogenation typically produces advantageous optoelectronic properties to improve the open-circuit voltage (*V*_OC_) and fill factor (FF) of devices but it narrows compatibility with acceptor materials, and thus increasing the overall SC. As researchers continue to find an optimal balance between SC and performance metrics, *reducing the number of synthetic steps is essential for lowering the complexity of conjugated polymers*. Furthermore, OPVs are not the only application where conjugated polymers suffer from complex syntheses. As such, the broader conjugated polymer community is needing to adopt more of the “12 Concepts of Green Chemistry”^17^ and serves as motivation for starting a new research group.

In recent years, there have been many approaches dedicated to simplifying the synthesis of conjugated polymers ranging from sustainable synthesis strategies (direct arylation polymerizations (DArP), acid-catalyzed condensations, etc.)^18^ to reducing the number of synthetic steps.^19,20^ However, each of these approaches still commonly involve multistep syntheses of monomers, tedious purifications (columns), use of hazardous materials, or low yields. As we were seeking ways to contribute to the conjugated polymer community, especially from an aspect of sustainability, we became aware of electron-rich pyrrolo­[3,2-*b*]­pyrroles where the synthesis was reported to be accomplished in air, from abundant chemical feedstocks (anilines and aldehydes), and purification was accomplished with simple vacuum filtrations.^21,22^ Furthermore, these molecules were shown to have incredible optoelectronic properties with systematic tunability, making them useful in an array of applications (Scheme 1). Interested readers are directed to a recent book chapter and review for a comprehensive overview of the chemistry and properties of molecular DHPPs.^23,24^ Despite the reported successes of molecular DHPPs, there had not been any reports of DHPPs being enchained in a polymer repeat unit, thus, their macromolecular properties were unknown. Furthermore, the possibility of accessing monomer building blocks amenable to polymerizations in a single step was incredibly lucrative. As such, we set out to design and synthesize DHPP monomers that would create a novel, yet simplified, class of conjugated polymers.

**Scheme 1:**
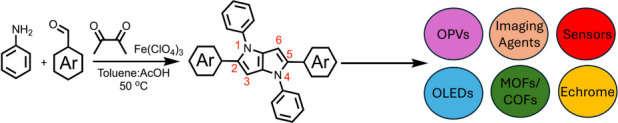
General Reaction for Accessing DHPPs and Examples of Applications

This broad overview is given to set the stage for how these concepts can be exploited for establishing a successful research program at a non-R1 university. Kleinschmidt and Lipomi have provided (written) context into the many important considerations that go into selecting and initiating research directions as a new PI and the importance of making the most of limited resources.^25^ However, since most PI’s receive graduate and postdoctoral training from research-intensive universities, young professors starting at R2-type universities may pursue research directions that are impractical for the infrastructure and experience levels of the students (likely mostly undergraduates). As such, we thought it could be impactful to share our group’s approach and experience when choosing a research direction at an R2 university.

Overall, the accessibility for the preparation of DHPP molecules and monomers offered the opportunity for researchers with limited experience to quickly gain confidence in the assembly, workup, and characterization of organic reactions. This would enable students to gain independence in a set of reactions that could be shared with other group members but also eliminate synthetic hurdles such that advanced characterization (NMR, UV–vis, doping, etc.) was enabled beyond the standard organic curriculum. The use of metal-catalyzed cross-coupling reactions that are ubiquitous in the field of conjugated polymers would enable exposure to contemporary polymerization protocols or molecular functionalization with specific tailorability. Combined, this simple exposure to aspects of green chemistry and next-generation electronic materials provided unique educational experiences to students while simultaneously contributing to the goal of reducing the synthetic complexity of conjugated polymers.

## DHPP-Based Polymers

Our initial goal was to synthesize a dihalogenated monomer so that the DHPP scaffold may be enchained in the polymer repeat unit via Pd-catalyzed cross-coupling polymerizations. While DHPPs had been shown to participate in molecular Pd-catalyzed cross-coupling reactions, such as Suzuki and Sonagoshira cross-couplings,^26–28^ we were motivated to maximize the impact of green chemistry and use DArP. The commercial availability of 4-bromobenzaldehyde and 4-decylaniline inspired the first design of a DHPP monomer such that reactivity in Pd-catalyzed polymerizations and solubility of the resulting copolymer were accounted for, respectively. Furthermore, we showed that we could attain 15 g of this monomer in a single step without sacrificing purity needed for efficient polymerizations (i.e., maintaining stoichiometric balance via Carothers equation) such that the simple chemistry is scalable. After proving the 3,6-protons of the DHPP scaffold were not sufficiently reactive under direct arylation conditions to introduce appreciable coupling defects,^29^ we reacted the Br_2_DHPP monomer with a dioctyl-dioxythiophene comonomer for the first example of a DHPP-containing copolymer with a number-average molecular weight (*M*_n_) ≈ 10.5 kg/mol attained via DArP.^1^ Importantly, the SC calculated with eq 1 was 13.8 which represented a reduced complexity to many conjugated polymers^14^ and in the range of current state-of-the-art “simple” conjugated polymers (*vide infra*).

Beyond the synthetic accomplishments, we were able to establish foundational structure–property relationships of DHPP-containing copolymers. There have been numerous studies focusing on the emissive properties of molecular DHPPs but we were focused on the potential redox properties of our synthesized polymers.^30^ Coupled with an electron-rich dioxythiophene, we were resolute that this was a worthy endeavor. As shown in Figure 1, the neutral polymer absorbed in the high-energy portion of the electromagnetic spectrum (EMS) to yield a yellow film. Upon oxidation, the absorbance profile transitioned to show two broad bands across the visible and NIR regions of the EMS, corresponding to formation of a black film. This gives the first example of a multicolored electrochromic DHPP material and motivated expanding the structural diversity of this family of polymers.

**Figure 1:**
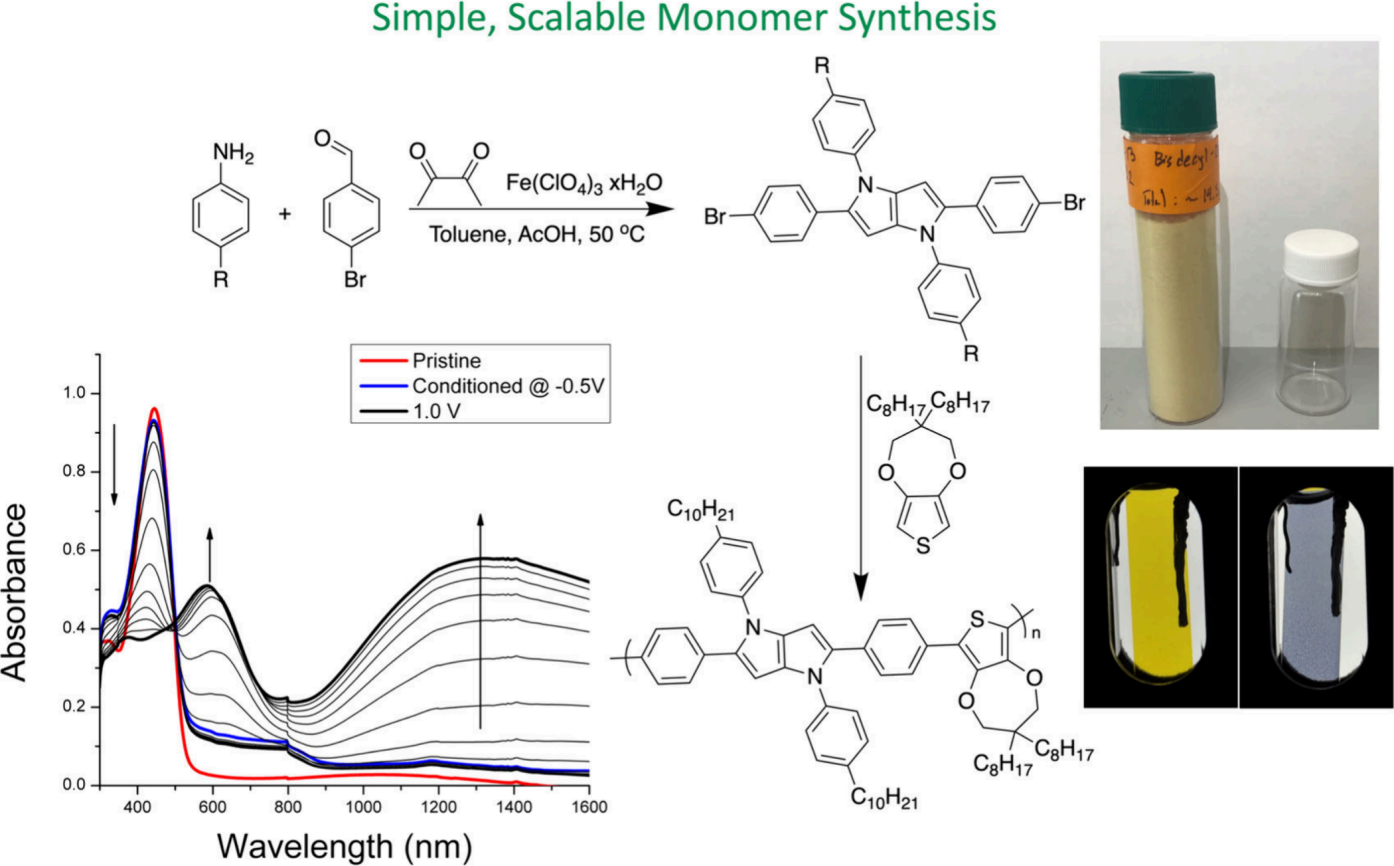
Synthetic strategies for scalable monomer synthesis and copolymerization to attain the first example of DHPP enchained in a polymer repeat unit. Characterization of the resulting polymer via spectroelectrochemistry and photography revealed yellow-to-black multicolored electrochromism. Images within this figure are adapted and reconfigured from ref 1.

The donor–acceptor approach is a robust strategy for manipulating the optoelectronic properties of conjugated polymers. The electron-rich nature of the DHPP scaffold further motivated this approach and served as the logical next step for expanding structure–property relationships of DHPP copolymers. However, initial efforts to accomplish this with our decyl-functionalized DHPP monomer were unsuccessful due to poor solubility of the resulting copolymers and measuring multimodal molecular weight distributions with high-temperature GPC. This required utilizing “new-to-DHPP” chemistries to install solubilizing side chains to the aniline starting materials.^31,32^ We accomplished this using another scalable chemical feedstock (acetaminophen) for the alkylation step followed by deprotection to attain customizable anilines in high yields (Figure 2(A)). Importantly, substituting the branched side chains on the ether functionalities did not inhibit attaining brominated DHPPs via the Fe-catalyzed MCR, which subsequently enabled optimizing DArP conditions with the electron-deficient comonomers (Th_2_DPP and TPD) in Figure 2(B).^3^ After careful optimization, *M*_n_’s up to ∼ 74 kg/mol were obtained using anhydrous DArP conditions while maintaining monomodal molecular weight distributions in the GPC traces.

**Figure 2:**
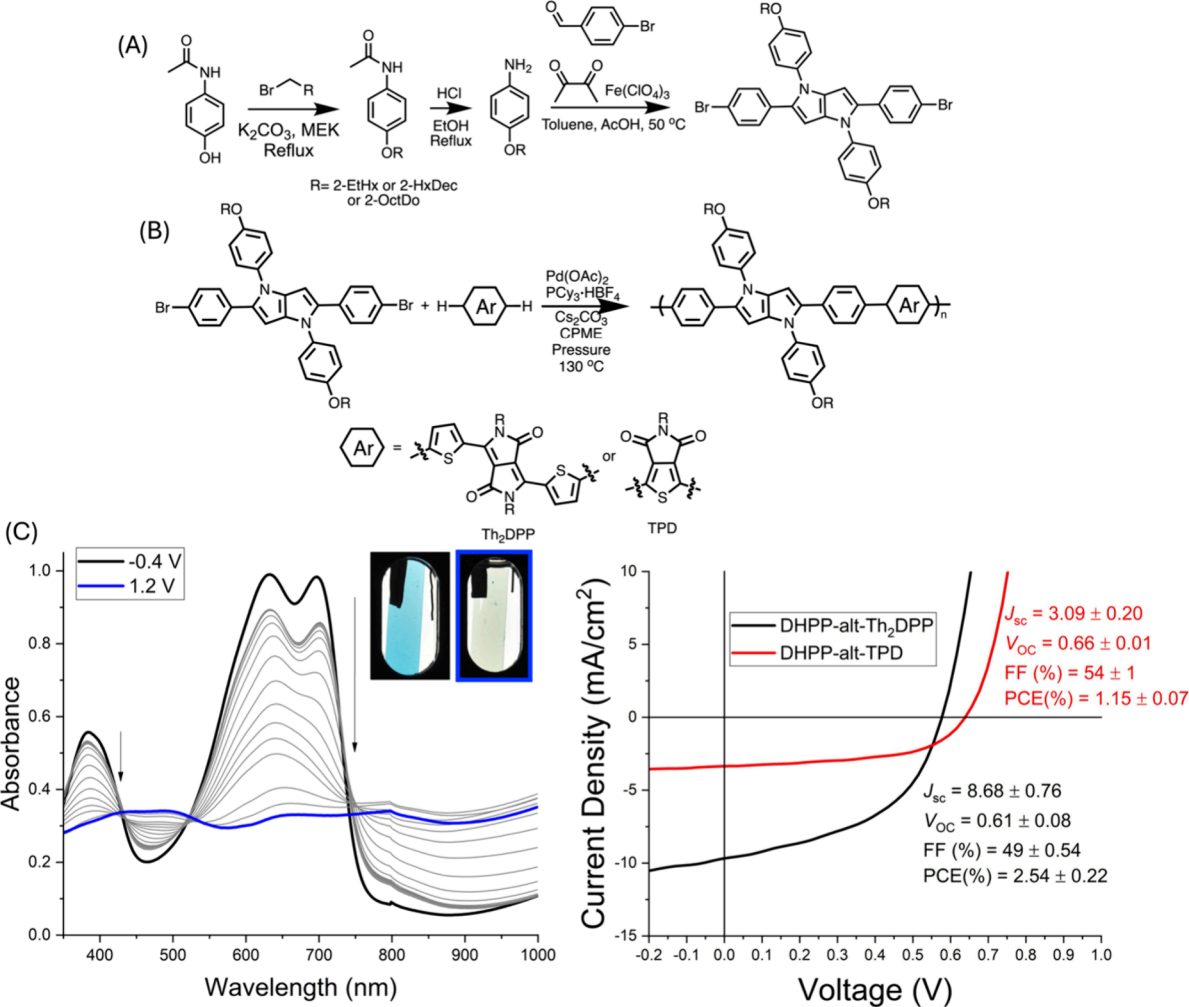
(A) Synthetic approach for synthesizing customizable anilines used in the synthesis of DHPP monomers followed by (B) DArP polymerization of DHPP with electron-deficient comonomers. (C) Spectroelectrochemistry and photography results reveal the first example of a colored-to-transmissive DHPP electrochromic polymer and photovoltaic response of donor–acceptor DHPP copolymers. Adapted in part with permission from ref 3. Copyright 2024 John Wiley and Sons Inc.

The improved solubility enabled solution processing of these polymers to probe their applicability in redox and solid-state applications. Spectroelectrochemistry experiments shown in Figure 2(C) show that the copolymer DHPP-co-Th_2_DPP displays a dual-band absorbance as a neutral film, common with donor–acceptor copolymers,^12^ and switches to a transmissive oxidized film with increasing electrochemical potential to represent the first color-to-transmissive DHPP electrochrome. This same polymer could be blended with fullerene or nonfullerene acceptor materials and obtain power conversion efficiencies (PCEs) ∼ 2.5%. The DHPP-co-TPD copolymer also served as a light-absorbing material in OPV devices to achieve PCEs ∼ 1.2%. While these results do not represent record-breaking OPV performance, the polymers did show 3–5× orders of magnitude increase in hole mobility compared to previously published molecular DHPPs.^33^ More specifically, average mobility (μ_avg_) values between 10^–6^ – 10^–4^ cm^2^V^–1^s^–1^ were obtained for both DHPP-co-Th_2_DPP and DHPP-co-TPD. These values are important because they fall in the range Blom and co-workers report to produce highest efficiencies for PCBM-based OPVs while the benchmark system of PM6:Y6 is measured to have hole mobilities ∼ 10^–4^ cm^2^V^–1^s^–1^.^34,35^ Combined, these results suggest that these polymers could achieve higher PCE values with further optimization of device processing and manufacturing parameters. Additionally, a deeper investigation into morphological effects and long-term stability is necessary to realize the full potential of these new systems.

Due to the many commercially available aldehydes, and the successes reported in the molecular literature where heteroaromatic scaffolds may be introduced to the DHPP periphery, we turned to using DHPP peripheral substituents to manipulate polymer optoelectronic properties. Here, we synthesized dibrominated phenyl, thienyl, and benzothiadiazole-functionalized DHPPs that were subsequently polymerized via Suzuki cross-coupling polymerizations (Figure 3).^5^ These polymerizations were also efficient, evident by *M*_n_’s ≈ 12–14 kg/mol and monomodal dispersities. The utility of varying aromatic units resulted in optical properties of the polymers being easily tuned across the entire visible spectrum. The thienyl and benzothiadiazole polymers showed red-shifted absorbances compared to the phenyl polymer due to increased planarity and charge-transfer excitations via the donor–acceptor approach, respectively. The redox stability with repeated electrochemical cycling was improved for the thienyl polymer compared to both the phenyl and benzothiadiazole polymers, evident by stable cyclic voltammograms for 400 cycles, which is important for continued development of these materials in redox applications. The thienyl-containing polymer also showed more efficient electrochemical doping during spectroelectrochemistry experiments, evident by the evolution of intense polaronic absorbance bands in the IR portion of the EMS with increasing electrochemical potential, similar to spectral features measured for P3HT. In contrast, the phenyl-containing polymer showed weak absorbance when electrochemically doped while benzothiadiazole did not display potential-dependent spectral changes.^36^ The manipulation of optical properties were also accompanied with excellent thermal stabilities (*T*_d_ > 400 °C). In total, this work expanded the structural diversity of DHPP-containing polymers and reinforces repeat unit dependence on polymer properties.

**Figure 3:**
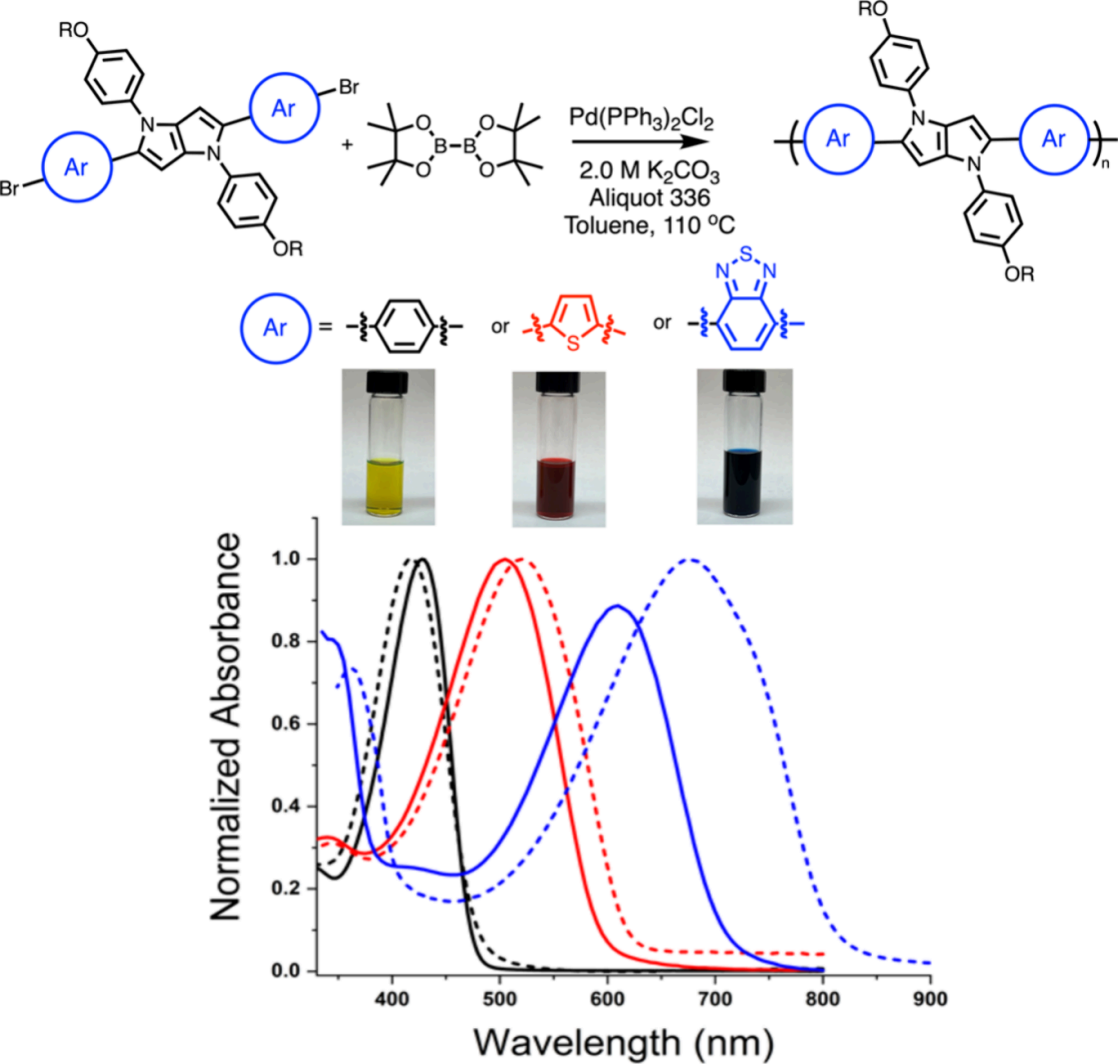
Simple modifications to the DHPP periphery enables new polymerization strategies and ability to tune optoelectronic properties across the entirety of the visible spectrum. UV–vis absorbance spectroscopy of polymer solutions (solid lines) and films (dashed lines) support the compositional-dependence of the polymer optical properties. Adapted in part with permission from ref 5. Copyright 2025 Royal Society of Chemistry.

While solving the synthetic complexity challenge is an important task for the conjugated polymer community, it is also necessary to be thinking about end-of-life considerations as well. Recently, researchers have been working to install labile linkages into the conjugated backbone so that systematic and controlled degradation is accomplished.^37^ Inspired to combine synthetic simplicity with sustainability/circularity, we synthesized a dialdehyde-functionalized DHPP that was susceptible to acid-catalyzed polymerizations with diamine monomers (Figure 4(A)).^33^ The diagnostic peaks of azomethines (imines), aldehydes, and DHPPs enabled molecular weight estimation via ^1^H NMR to be ≈ 5.4 kg/mol, which corresponds to approximately 20 rings (5 repeat units) and exceeding the effective conjugation length. These same diagnostic peaks also enable monitoring degradation in the presence of trifluoroacetic acid (TFA).^2^ As shown in Figure 4(B) and (C), the DHPP polymer rapidly degrades and this degradation is accompanied with quantitative spectroscopic changes in absorbance and fluorescence as well as qualitative color changes and a bright fluorescence “turn on” effect. These results spurred a systematic study on environmental effects on the degradation of imine-containing polymers where the degradation is shown to be solvent dependent and degradation rates increase with increasing acidity.^38^ Another notable feature from these studies is the ability to recover monomer building blocks with identical structural integrity when compared to as-synthesized monomers (Figure 4(D)), thus emphasizing the potential circularity of these materials. Combined, these efforts represent expanding the structural diversity of degradable conjugated polymers with a synthetically simple building block and relating important environmental influences to the eventual degradation. In addition to recyclability efforts, this knowledge will also be important to fields such as drug delivery or biosensing/imaging.

**Figure 4:**
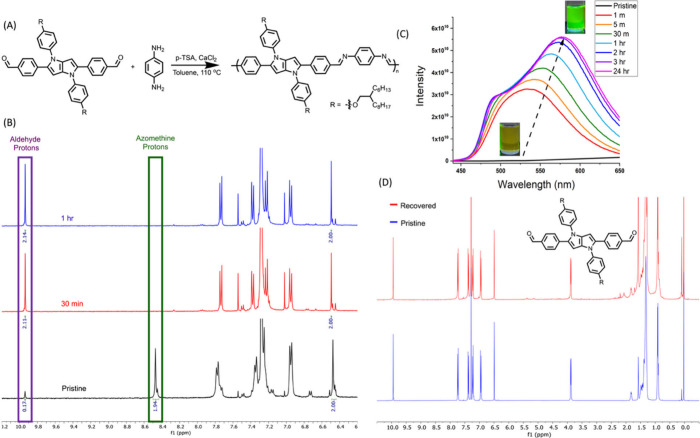
(A) Polymerization of a dialdehyde DHPP monomer via acid-catalyzed polycondensation to attain an azomethine-containing copolymer. (B) ^1^H NMR spectra monitoring and confirming the conversion of azomethine linkages to aldehydes after exposure to acid to reveal rapid and complete degradation. (C) Evolution of emission measured via fluorescence spectroscopy during degradation and the intense fluorescence color change. (D) ^1^H NMR of as-synthesized and recovered dialdehyde DHPP after degradation, emphasizing prospects of recyclability. Adapted in part with permission from ref 2. Copyright 2023 John Wiley and Sons Inc.

## Take-Home Message from Polymerizations

As device performance metrics continue to increase, synthetic complexity remains a challenge needing to be addressed in the conjugated polymer community. DHPPs offer a new building block both as “homopolymers” and copolymers that, based on the quantitative standard of the field at this time, reduces the SC and does not relinquish the tailorability of optoelectronic properties. The immediate advantage is the reduction of synthetic steps of the monomers used for polymerizations, minimized purification protocols, and reduced use of hazardous materials. The plethora of commercially-available aldehydes and synthetic addressability of aromatic building blocks will likely enable unrealized attributes in the future. It is important to note that researchers should be mindful of possible adjustments to purification protocols or reduced yields when changing peripheral aromatic units that influence the synthetic complexity. For example, the thienyl-DHPP monomer was isolated after reprecipitation while the benzothiadiazole-DHPP monomer required recrystallization. The synthetic “simplicity” of DHPP polymers is emphasized in Figure 5, where SCs of polymers described in the Account are compared to legacy (P3HT, ProDOT) and state-of-the-art conjugated polymers (PTQ10, PM6). Furthermore, the reduction of synthetic steps results in DHPP monomers having scalability factors (SFs) calculated in the range (6 ≤ SF_DHPP_ ≤ 14) similar to the high-performing thiophene donors (3 ≤ SF_Th_ ≤ 18) analyzed by Marks and co-workers. While DHPPs are not the “most simple” conjugated polymers, SCs are commonly reduced by ∼ 35% compared to analogous donor–acceptor systems (DPP, TPD) when incorporating DHPP as a monomer building block. The simplicity of DHPP polymers is envisioned to continue to improve as monomer yields are further optimized. Also, DHPPs minimize numerous synthetic challenges, such as the use of moisture sensitive Grignard reagents or production of toxic waste via Stille polymerizations.

**Figure 5:**
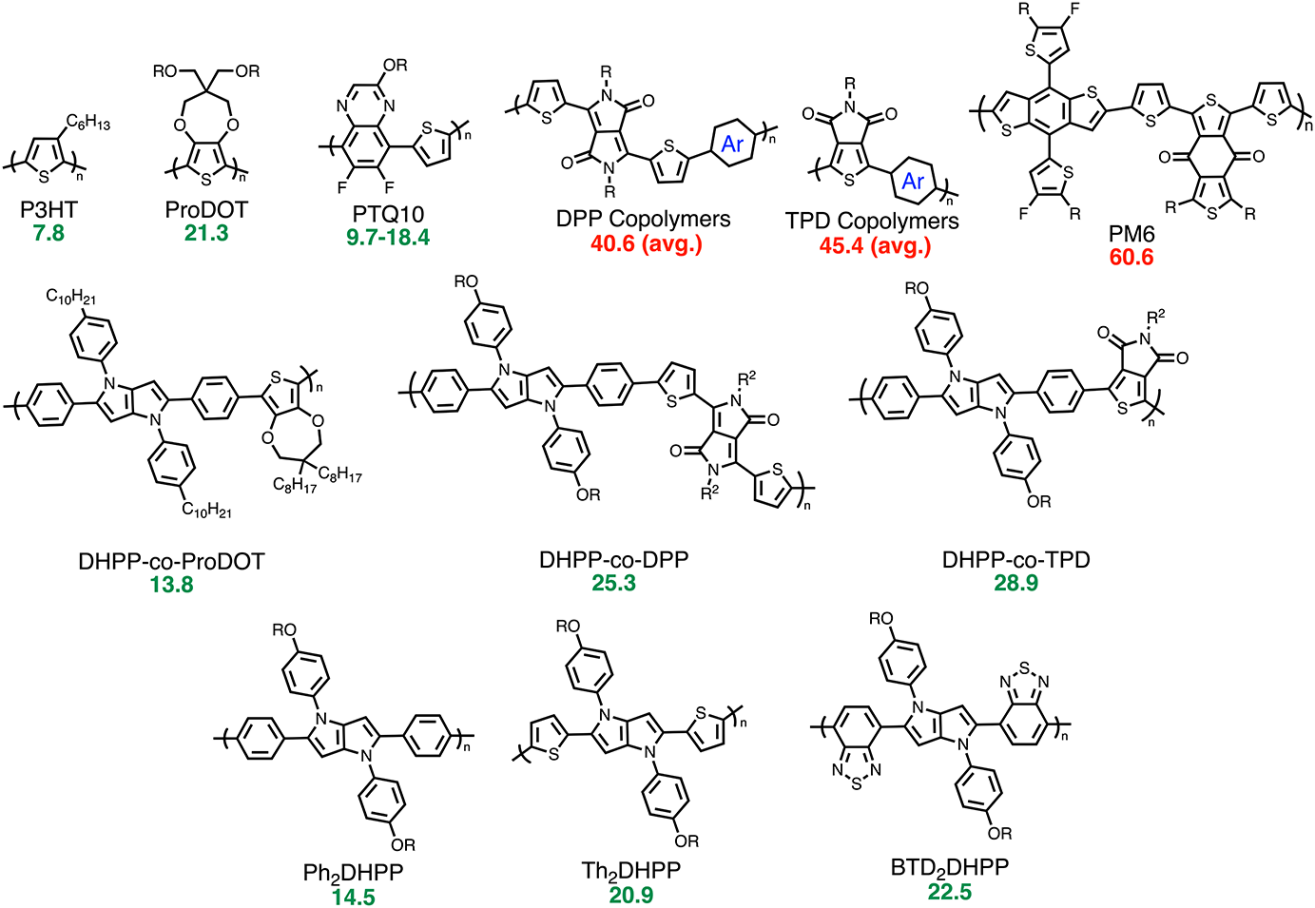
Representative structures of legacy conjugated polymers and DHPP-containing polymers accompanied with their calculated synthetic complexities.

The propensity for DHPPs to participate in numerous polymerization protocols (Scheme 2) enables attaining and tuning desired macromolecular properties. It is not unreasonable that these same scaffolds would be amenable to analogous metal-catalyzed polymerizations (Stille, Sonagoshira, etc.)^39,40^ that would expand the structural design space of the DHPP polymers for new applications. The demonstration of using chemistries to install customized side chains pave the way for relating side chain functionality to polymer properties and phase behavior.^41–44^ Expanding the side chain engineering approaches for application-specific functionality, pursuing targeted molecular weights via reaction optimization, and expanding the comonomer design space remain worthy research pursuits for this underutilized class of conjugated polymers. The continued tailorability of DHPP polymers is emphasized in Figure 6, where ester-functionalized DHPPs are susceptible to hydrolysis to generate water-soluble polymers or hydrophilic polymer films.^45–47^ This is an example of the tailorability that inspires continued development of DHPP polymers and what may be unknown properties of conjugated polymers enabled from a tailorable building block.

**Scheme 2:**
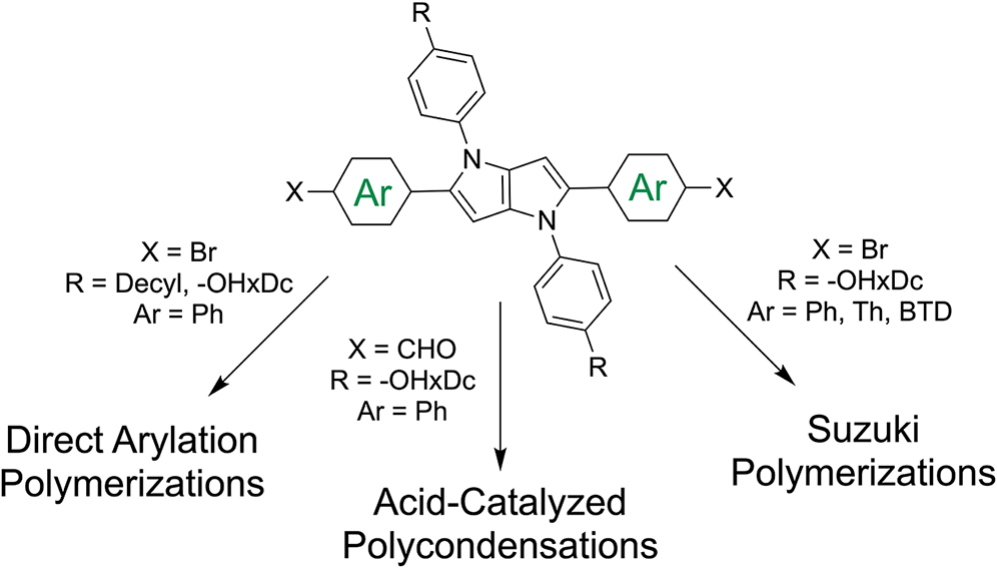
Examples of Polymerization Protocols Amenable for Synthesizing DHPP-Containing Polymers

**Figure 6:**
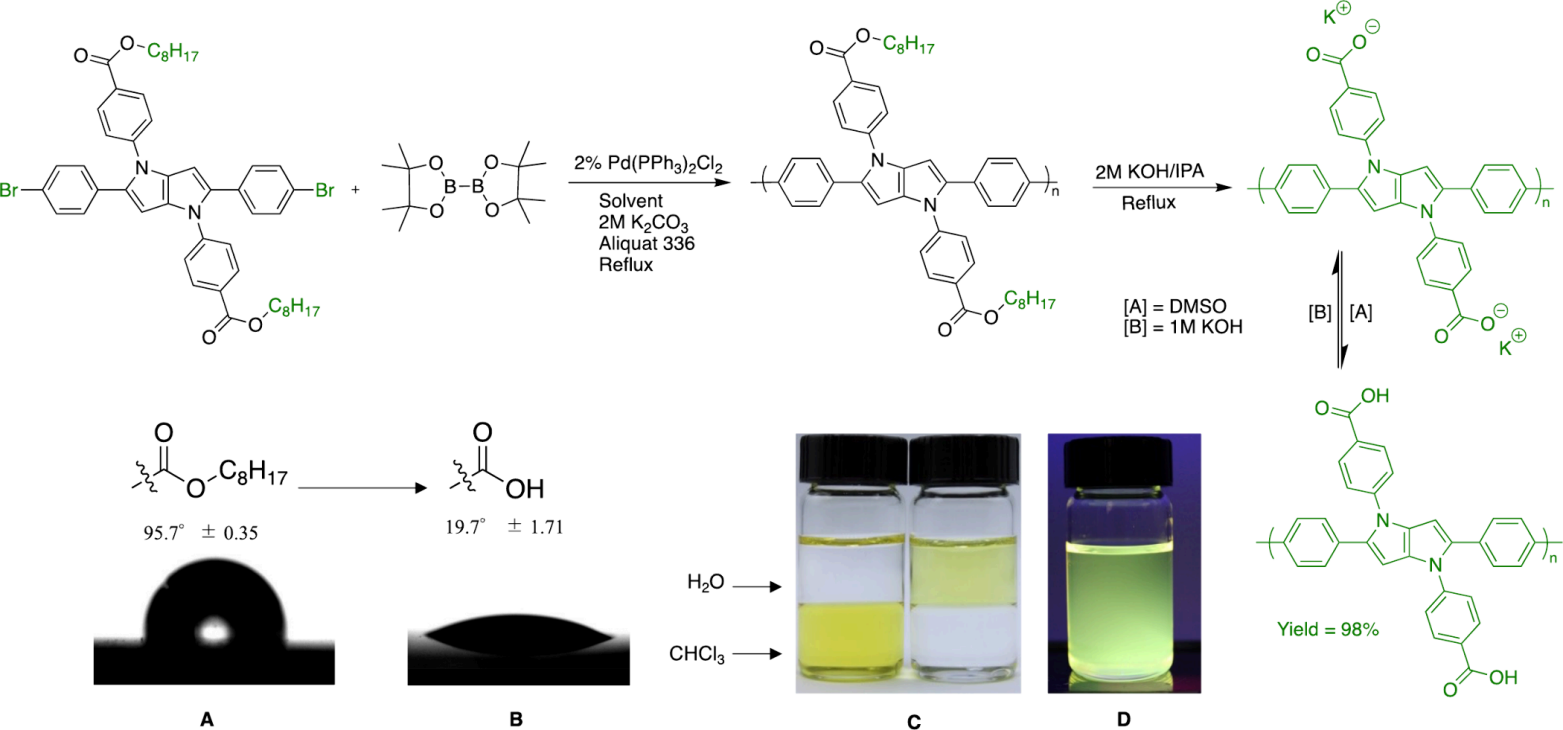
Synthesis of an ester-functionalized DHPP polymer and subsequent saponification to access water-soluble conjugated polymers.

## DHPPs as High-Contrast Electrochromes

While we demonstrate the utility of DHPPs to simplify the synthesis of conjugated polymers, their utility in electrochromic and redox applications is in a nascent state. The ability of DHPPs to participate in Pd-catalyzed cross-coupling reactions also enables constructing molecular scaffolds to probe how precise installation of coupling partners with varying electronic configurations affects optoelectronic properties of neutral and oxidized species. From a simplistic approach, we wanted to understand the “yellow-to-black” electrochromism observed with our first-generation DHPP copolymer.

Our approach started with performing Suzuki cross-coupling reactions with various aryl boronic acids to install 1 additional aromatic unit to each side of the DHPP scaffold. We hypothesized that subtle changes to peripheral substituents would ultimately enable a systematic study on substituent effects of DHPP chromophores as neutral and oxidized molecules. As shown in Figure 7(A), and similar to our polymer efforts, DHPPs may be functionalized with a variety of aromatic substituents to create a large family of π-extended DHPPs.^48^ While our work narrowed in on 5 different chromophores, highlighted here in Figure 7(B) are 2 extremes of electron-withdrawing and -donating effects (cyano vs methoxy). The cyano-functionalized DHPP has a neutral absorbance that extends into the visible portion of the EMS due to the push–pull nature of the dye brought on the by the electron-rich DHPP and electron-deficient cyano group. However, when the push–pull nature of the dye is suppressed by utilizing electron-rich substituents on the periphery of the chromophore, the neutral absorbance shifts mostly to the UV portion of the EMS (solid lines, Figure 7(C)). The peripheral substituents also had distinct influence on the radical cation absorbances (dashed lines in Figure 7(C)), with the cyano group displaying a dual-band absorbance profile across the visible spectrum while the methoxy-functionalized chromophore has a more red-shifted radical cation absorbance.

**Figure 7:**
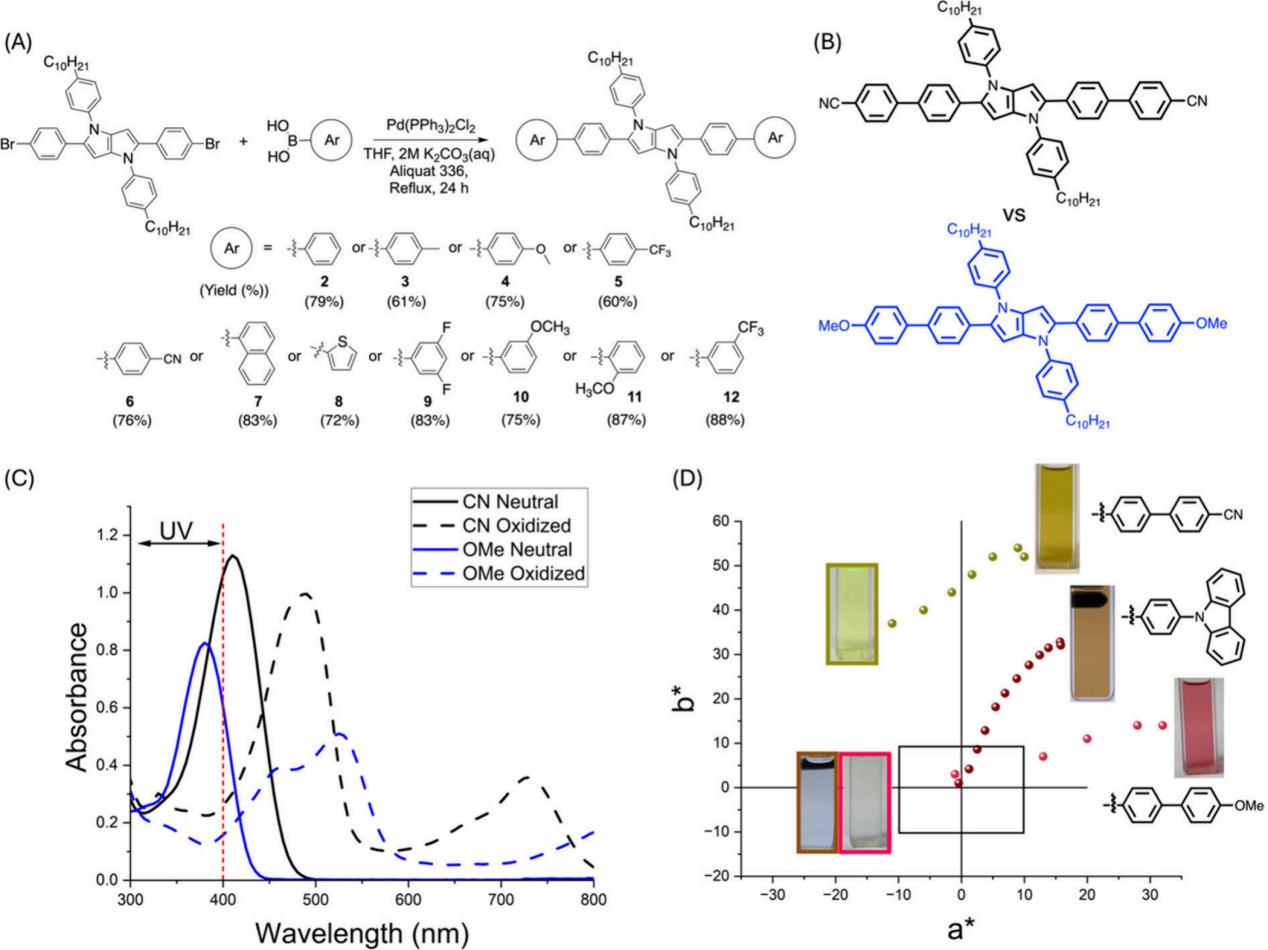
(A) Suzuki cross-coupling reactions demonstrating the expansive design space for π-extended DHPP chromophores. (B) Representative structures for electron-withdrawing (black) and electron-donating (blue) chromophores used to compare optical properties in (C). (D) Color coordinate plots and corresponding photographs illustrating color changes induced by oxidative doping of DHPP solutions via titration with Fe­(ClO_4_)_3_.

The measured absorbance differences ultimately manifest themselves as distinctly different colors qualitatively (visually) and quantitatively when converted to L*a*b* color coordinates (Figure 7(D)). First, the cyano-functionalized DHPP starts as a vibrant, highlighter yellow color in the neutral state that switches to a saturated gold color upon oxidation, which is due to absorbance spectra of neutral and oxidized molecules spanning across the visible spectrum. The diminishment of the push–pull phenomenon of the methoxy-functionalized DHPP results in a highly transmissive, color-neutral solution that transitions to a burgundy red color upon oxidation. The box on the color space plot in Figure 7(D) represents the defined space for color neutrality (a*b* = ±10)^49^ and chromophores that possess these transmissive-to-colored switches upon oxidation are known as anodically coloring electrochromes (ACEs).^50^ With the knowledge that ACE DHPPs are possible, we gained essential insight into foundational structure–property relationships that enable manipulating neutral and oxidized absorbances, and ultimately color, to attain a novel class of high-contrast electrochromic materials. More recently, we expanded this utility of π-extended DHPPs through the synthesis of a carbazole-terminated DHPP.^51^ This chromophore also absorbs mostly in the UV portion of the EMS and transitions to the visible portion to yield a vibrant brown oxidized molecule and emphasizes the ability to systematically tune the resulting color of our materials.

After learning how to manipulate optoelectronic properties of the π-extended DHPPs, we envisioned DHPPs would enable the elimination of Pd-catalyzed cross-coupling reactions to construct high-contrast ACE molecules while simultaneously being a modular scaffold for systematic color control. We turned to theory to support the belief that subtle changes of peripheral substituents would facilitate the desired neutral absorbances in the UV and manipulation of the radical cation to achieve color control.^52^ An example of these results is presented in Figure 8(A) where fluorinated and methoxy-functionalized DHPPs are predicted to absorb in the UV portion of the EMS while the positioning of the radical cation absorbance is affected by the electronic nature of the peripheral substituents. We surveyed several molecules via time-dependent density functional theory (TD-DFT) that ultimately guided our synthetic efforts in the laboratory to attain a family of chromophores with varying electronic functionalities.^4^

**Figure 8:**
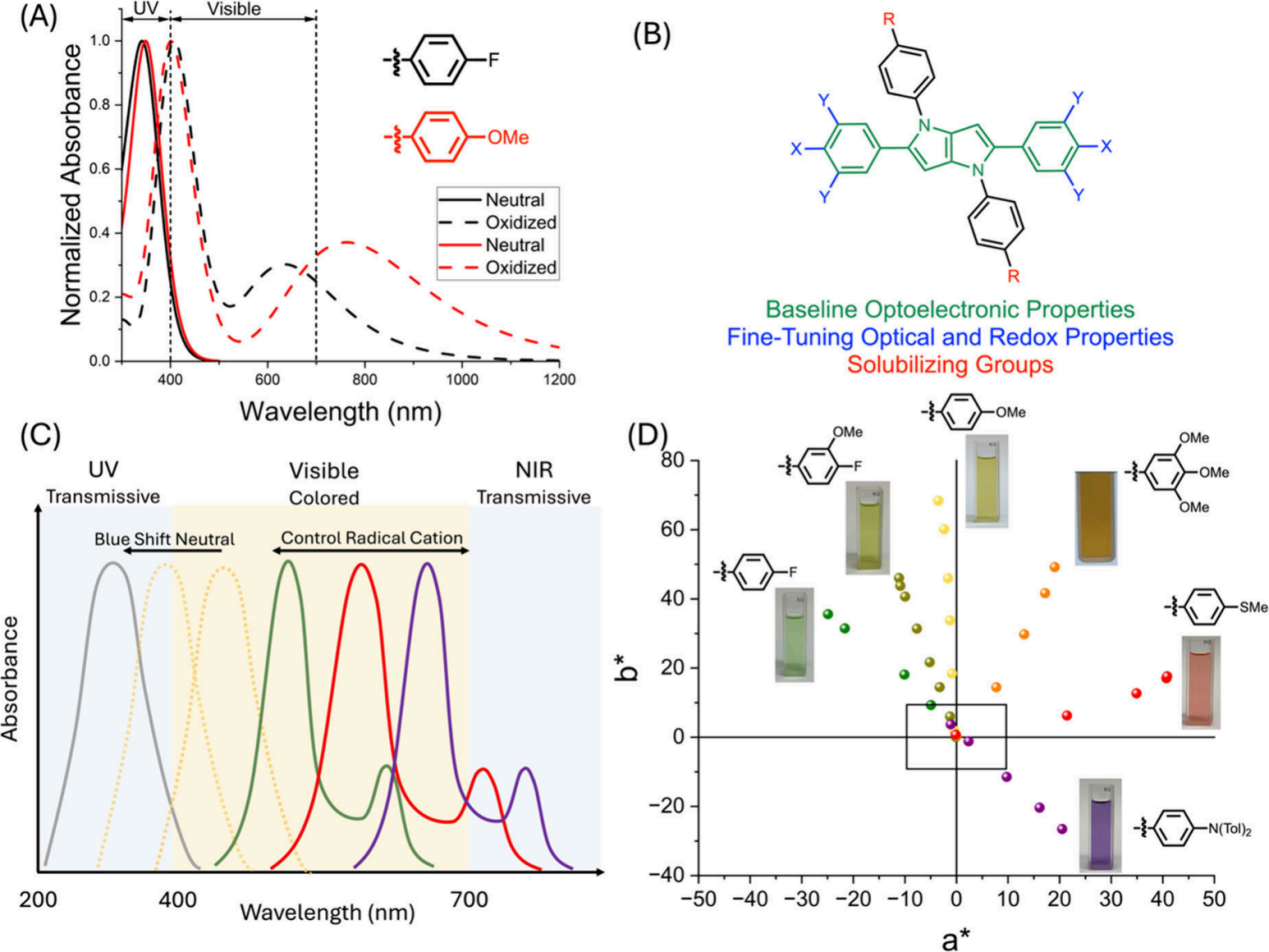
(A) Simulated absorbance spectra of neutral and oxidized DHPPs calculated using TD-DFT. (B) Molecular design paradigm for manipulating optoelectronic properties of DHPP chromophores as neutral and oxidized materials. (C) Illustration showing the design paradigm in practice to position neutral absorbance in the UV while enabling systematic control of radical cation absorbances. (D) Color coordinate plot demonstrating the utility of the DHPP design paradigm to attain a family of highly transmissive neutral molecules that transition to precise colors across three quadrants of the L*a*b* color space with the accompanying photographs.

This approach now allows us to teach design-structure–property relationships using the generic design paradigm in Figure 8(B). Here, the DHPP core and the 2,5-functionalized aromatics provide the baseline optoelectronic properties for the family of electrochromes. Peripheral functionalization along the 2,5-axis enables fine-tuning of redox and optical properties while R-groups along the 1,4-axis enable sufficient solubility for thorough characterization. These considerations lead to the optical features illustrated in Figure 8(C). Here, diminishing the push–pull nature of the chromophores blue shifts the neutral absorbance into the UV to obtain highly transmissive solutions (L*a*b* = 100, 0, 0). Upon oxidation, the positioning and shape of the radical cation absorbance is dictated/manipulated by the 2,5-peripheral substituents. As the electron-donating ability of these functional groups is increased, the neutral absorbance remains in the UV while the radical cation is systematically red-shifted across the visible spectrum. These structural controls are most apparent in the colorimetry and photographs presented in Figure 8(D), where colors of oxidized solutions are attained across 3 separate color quadrants while remaining highly transmissive in the neutral state (a*b* ≈ 0,0). Our results position DHPPs to have comparable contrast and color control to dioxythiophene-based ACE molecules^53–56^ while simultaneously eliminating the need for Pd-catalyzed cross-coupling reactions or multistep syntheses. Furthermore, we envision mimicking strategies similar to Mei and co-workers^57^ will enable solution processing of DHPP electrochromic polymers as thin films and testing their applicability in devices.

## Conclusions and Outlook

The design space for conjugated materials is arguably only limited by a researcher’s imagination and synthetic capabilities. However, there has to be a greater intentionality for using simple and scalable chemistries to advance the field beyond studying intriguing structures on small, laboratory scales. This Account highlights contributions to these considerations with the theme of exploiting the robust synthesis of DHPPs to simplify the complex synthesis commonly associated with conjugated polymers without sacrificing the ability to control optoelectronic properties. Polymers synthesized using DHPP (co)­monomers are calculated to quantifiably reduce the synthetic complexity compared to many state-of-the-art conjugated polymers while having design-specific properties and functionality. These polymers have found utility in polymer-based OPVs and color-changing (electrochromic) films. Labile linkages may also be incorporated into these polymers so that the resulting materials may be degraded on demand for circularity/recycling or potential bioimaging and therapeutics. In regard to electrochromic phenomenon of DHPPs, the plethora of cost-effective starting materials has facilitated development of a design paradigm that enables “spectral engineering” of radical cation absorbances, and thus systematic color control.

It is clear that DHPP is a versatile building block for the design and synthesis of new conjugated materials but there is a compelling case that there is a need for further design strategies. The robust side chain chemistry will enable installation of functionalities, such as charge or H-bonding motifs, that will be useful in controlling other properties beyond solubility (i.e., phase behavior, self-assembly morphology, etc.). Merging new side chain engineering approaches with degradability is also compelling and would reveal potentially new structure–property relationships. Finally, accessing the fourth color quadrant for the ACE molecules is of significant interest and would elevate color control of DHPP electrochromes into the company of dioxythiophene-based polymeric electrochromes.

The modular approach to DHPP design and synthesis has produced abundant opportunities for dedicated mentoring and teaching in a laboratory setting. Of the papers published since our group’s inception in 2020, and described in the Account, undergraduates have substantially contributed to experimental design, data collection, and presentation to earn authorship on each one. Most former students have continued their education in graduate school or found employment in a STEM field. These metrics further cement DHPPs as a useful building block for optoelectronic materials through the training of future scientists by “keeping it simple”. It is my hope that the work described here further inspires others and is instructive to early career faculty choosing research directions in their respective fields.
